# Degradation of okadaic acid in seawater by UV/TiO_2_ photocatalysis – Proof of concept

**DOI:** 10.1016/j.scitotenv.2020.139346

**Published:** 2020-09-01

**Authors:** Dolores Camacho-Muñoz, Linda Ann Lawton, Christine Edwards

**Affiliations:** School of Pharmacy and Life Sciences, Robert Gordon University, Aberdeen AB10 7GJ, UK

**Keywords:** Okadaic acid, Seawater, Photocatalysis, Mass spectrometry

## Abstract

The consumption of contaminated shellfish with marine toxins causes adverse socioeconomical, environmental and health impacts. The marine toxin okadaic acid (OA) provokes diarrhetic shellfish poisoning (DSP) syndrome characterized by severe gastrointestinal symptoms. Therefore, there is increasing interest in removing these toxins from the marine environment to protect shellfish harvesting sites. Photocatalysis is proposed as an efficient method to detoxify the marine environment.

In this study, *Prorocentrum lima* was used to produce high purity DSP toxins, in particular OA, for degradation studies. The profiling, characterization and quantification of DSP toxins in the culture of *P. lima* were achieved by ultrahigh performance liquid chromatography coupled to quadrupole-time of flight mass spectrometry (UPLC-QTOF-MS^E^) for accurate-mass full spectrum acquisition data. The effectiveness of UV/TiO_2_ system to degrade OA in seawater was assessed in lab-scale experiments and identification of transformation products was proposed based on the data obtained during analysis by UPLC-QTOF-MS^E^. The detoxification potential of the UV/TiO_2_ system was investigated using the phosphatase inhibition assay.

Sufficient amount of high-purity OA (25 mg, >90% purity) was produced in-house for use in photocatalysis experiments by simple reversed-phase flash chromatography. Complete degradation of OA was observed in seawater after 30 min and 7.5 min in deionized water. The rate constants fitted with the pseudo-first order kinetic model (R^2^ > 0.96). High-resolution mass spectrometry analysis of the photocatalyzed OA allowed tentative identification of four transformation products. Detoxification was achieved in parallel with the degradation of OA in deionized water and artificial ocean water (≤20 min) but not for seawater. Overall, results suggest that UV/TiO_2_ photocatalysis can be an effective approach for degrading OA and their TPs in the marine environment.

To the best of our knowledge, this is the first report on the use of photocatalysis to degrade marine toxins and its promising potential to protect shellfish harvesting sites.

## Highlights

• High purity okadaic acid produced in-house from *P. lima* by simple reversed-phase flash chromatography.
• UV/TiO_2_ applied to the degradation of the marine toxin, okadaic acid.
• Okadaic acid was degraded by UV/TiO_2_ in 30 min in seawater.
• Transformation products were characterized by UPLC-QTOF-MS^E^.
• Reduction in toxicity after UV/TiO_2_ was demonstrated by protein phosphatase inhibition assay.

## Graphical abstract

**Figure:**
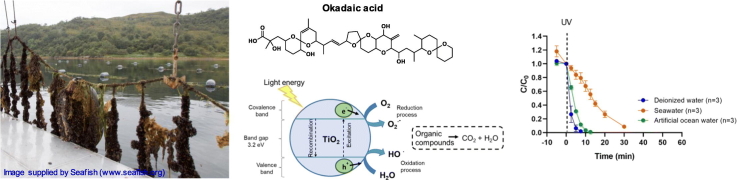

## 1 Introduction

Marine toxins are produced by natural phytoplankton and can be accumulated by filter feeding shellfish, meaning that they may enter the food chain and cause toxic episodes in human health. Marine toxins are responsible for >60,000 intoxications per year, with overall mortality of around 1.5% (Kantiani et al., 2010). Based on their resultant illness, marine toxins can be classified in diarrheic shellfish poisoning (DSP) toxins, paralytic shellfish poisoning (PSP) toxins, amnesic shellfish poisoning (ASP) toxic, neurotoxic shellfish poisoning (NSP) toxins, ciguatera fish poisoning (CFP) and clupeotoxin fish poisoning (CLP) toxins (Farabegoli et al., 2018; Gerssen et al., 2010).

The dinoflagellates *Prorocentrum* (*P. fortii*, *P. lima*, *P. concavum* and *P. minimum*) and *Dinophysis* (*D. fortii*, *D. acuminate*, *D. acuta*) are the main producers of DSP toxins (Aquino-Cruz et al., 2018; Bravo et al., 2001; Lee et al., 2016; Luo et al., 2017; Nishimura et al., 2019; Pan et al., 2017; Uchida et al., 2018). Okadaic acid (OA) and dinophysistoxin 1 and 2 (DTX1, DTX2) are the original DSP toxins. Later on, DTX3 and the 7-O-acylated derivatives of OA and DTX were included as DSP toxins. These analogues are not produced by the dinoflagellates but they are formed in the bivalves (Rossignoli et al., 2011; Suzuki et al., 1999; Vale, 2010). OA has also been widely used in biochemistry research because it is an effective specific inhibitor of Ser/Thr protein phosphatase and consequently, cause cell cycle alterations, apoptosis and cytoskeleton disruption. In addition, OA exhibits tumour promoting activity, embryotoxicity and neurotoxicity (Cohen et al., 1990; Dounay and Forsyth, 2002; Munday, 2013).

In order to minimize economical loss and to protect human health caused by annual DSP outbreaks, monitoring systems have been implemented by local governments. They include the analysis of toxin content in shellfish, the concentration/presence of toxic algae in the water body and the evaluation of hydrographic/meteorological data (Anderson et al., 2019; Campbell et al., 2011; Shen et al., 2012). A current European regulatory limit of 160 μg of OA equivalents per kg of edible shellfish must not be exceed and this limit is currently accepted in several other countries (e.g.: USA, China, Brazil) (EU, 2002/225/EC). Liquid chromatography coupled with mass spectrometry detection (LC-MS) is the technique of choice for the analysis of DSP at trace levels in complex matrices (Dell'Aversano and Tartaglione, 2017). Although major advances have been made in the development of multi-residue analytical methods, unavailability of suitable standards, the high cost of the commercial standards, the presence of several toxin analogues and unknown toxic compounds are an additional challenge.

OA has been detected in nearshore seawater across the globe at concentrations in the ng/L range (Bosch-Orea et al., 2017; Fux et al., 2009; Hattenrath-Lehmann et al., 2018; He et al., 2020; Reguera et al., 2014). Even high concentrations of OA up to 1.78 μg/L in seawater and up to 560 μg/g in particulate matter were reported (Bosch-Orea et al., 2017). OA proved to be very stable in seawater, interstitial water and sediment (Blanco et al., 2018; Pizarro et al., 2009), thereby seriously threatening aquaculture industry and human health. Because of this, it was hypothesized that high concentrations of OA in seawater would indicate recent or past blooms that could last even a year (Blanco et al., 2018).

There has been an increased interest in finding suitable techniques to remove these toxins from the marine environment. Advanced oxidation processes (AOPs) are widely used to degrade organic pollutants through free radicals as reviewed by (Ibhadon and Fitzpatrick, 2013; Matafonova and Batoev, 2018). Of different AOPs, heterogeneous photocatalysis with TiO_2_ has been demonstrated to be an efficient method to remove freshwater toxins (e.g. cyanotoxins) (Antoniou et al., 2018; Liu et al., 2009; Pestana et al., 2015; Pinho et al., 2015). Major improvements have overcome the barriers to the application of this technology to complex matrices. For example, the feasibility of UV-LEDs as an alternative low energy source in complex environments (Schneider et al., 2019), the use of solar light activated materials to reduce application costs (Kinley et al., 2018), the addition of oxidants to reduce energy requirements (Antoniou et al., 2018) or the form of the catalytic material to recover it after treatment (e.g. fixed to a film, coated surfaces) (Kinley et al., 2018; Pestana et al., 2015; Shephard et al., 2002). However, this is still an understudied area regarding marine toxins (Djaoued et al., 2008; Khan et al., 2010).

This work aimed i) to produce cost effective amounts of high-purity DSP toxins from *P. lima* for photocatalysis experiments; ii) to evaluate the performance of a UV/TiO_2_ system to degrade/mineralize OA in seawater; iii) to identify possible transformation products (TPs) generated during photocatalysis; iv) to evaluate the detoxification efficiency of UV/TiO_2_ system for OA in seawater.

## 2 Materials and methods

### 2.1 Chemicals and reagents

LC-MS grade acetonitrile, methanol and formic acid were purchased from Sigma-Aldrich (Irvine, UK). Deionized water (DW) was provided by a Milli-Q system (Millipore, Watford, UK). TiO_2_-P25 from Degussa (Germany) was used as catalyst. It is a mixed phase containing ≈90% anatase and ≈10% rutile with a specific surface area of 50 m^2^/g. OA (4.2 μg in 0.5 mL ampoule) and DTX1 (4.2 μg in 0.5 mL ampoule) standards for confirmation purposes were obtained from National Research Council Canada (Ontario, Canada) (Fig. S1). Instant ocean salt was obtained from Aquarium systems (France). SNAP C18 120 g cartridges were bought from Biotage (Hengoed, UK). Cellulose acetate centrifuge tube filters (0.45 μm), *p*-nitrophenyl phosphate (pNPP) and the protein phosphatase-1 catalytic subunit (PP1c) from rabbit (specific activity 5000–15,000 U/mg protein) were supplied by Sigma-Aldrich (Irvine, UK).

### 2.2 Extraction and purification of DSP compounds from *P. lima*

*P. lima* CCAP 1136/11 (Culture Collection for algae and Protozoa, Oban, Scotland) was grown on seawater enriched with f2-Si medium (10 × 4 L medium in 10 L round Pyrex flasks; Guillard and Ryther, 1962) at 22 ± 1 °C with continuous light at 20 μmol/m^2^/s. Cultures were sparged with sterile air at 2.3 L/min and grown for 35 days (Praptiwi, 2014). Harvesting was achieved by sedimentation of cells and removal of the supernatant, concentrated cells were stored at −20 °C. Concentrated frozen cells obtained from 240 L cultures (approx. 5 × 104 cells/mL) were defrosted and combined to give a concentrated cell slurry (2 L). This cell slurry was combined with methanol and water at a ratio 1:1:3 (*v*/*v*/v) (culture-methanol-water) and loaded onto a SNAP C18 120 g cartridge (Biotage) to concentrate the toxins after tangential flow filtration (Biotage, Hengoed, UK). This process was repeated twice with the cell residue. Toxins were fractionated using a Biotage Selekt Flash Purification System (Biotage, Hengoed, UK). The mobile phase had a flow rate of 30 mL/min and a linear gradient from 10 to 100% MeOH over 90 min. Fractions (100 mL) were automatically collected using UV detection (220–254 nm). DSP content in each fraction was analysed by ultrahigh performance liquid chromatography coupled to quadrupole-time of flight mass spectrometry (UPLC-QTOF-MS^E^). OA containing fractions were pooled, dried down in a centrifugal evaporator (Genevac, Ipswich, UK) and stored at −20 °C.

### 2.3 Stability study

Aliquots of OA (10 μg/mL), isolated from the *P. lima* culture, were prepared in methanol and in methanol/water (1:1, v/v) and kept at −20 °C, 4 °C and room temperature (20–22 °C). Effect of high temperature (45 °C) and evaporation was also tested in an additional aliquot of OA (10 μg/mL). Samples were analysed by UPLC-QTOF-MS^E^ at day 0, 7, 14, 21, 28, 35 and 42.

### 2.4 Photocatalysis study

**Fig. 1:**
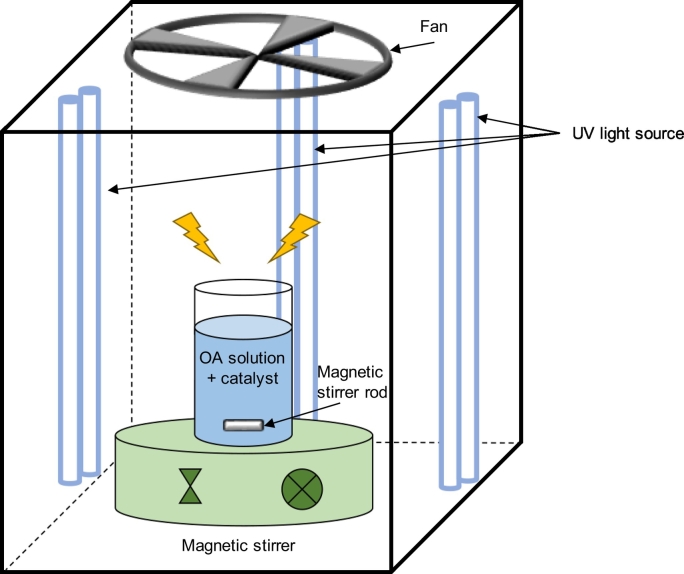
Schematic diagram of the photocatalytic reactor used in this study. UV light source at the front is not shown in the diagram for better visualization.

Photocatalysis experiments were conducted in a lab-scale photoreactor (Fig. 1) equipped with 4 UV lamps (Philips PL-L 36W/09/4P UVA; wavelength 315–380 nm). 10 mL of filtered deionized water (DW), artificial ocean water (AOW) and seawater (SW) (collected in May 2019 from Stonehaven, Aberdeenshire, UK) spiked with OA (10 μg/mL prepared in DW), isolated from the *P. lima* culture, were maintained under magnetic stirring at 20–22 °C for 60 min. Experiments were run in triplicate in the dark and under UV irradiation in the presence and absence of the catalyst TiO_2_ (0.1%, *w*/*v*). The experiments were performed at pH 6.9 in DW, 7.3 in AOW and 7.8 in SW. The suspension was kept in the dark under stirring during 5 min (adsorption-desorption phase) and afterwards the UV lamps were switched on. At selected time intervals (5 min before UV, 0, 2.5, 5, 7.5, 10, 12.5, 15, 20, 30 and 60 min) samples (0.5 mL) were collected and filtered through a 0.45 μm filter before UPLC-QTOF-MS^E^ analysis.

### 2.5 Analysis by UPLC-QTOF-MS^E^

OA and related compounds were quantified by UPLC-QTOF-MS^E^ (Waters, UK) equipped with an electrospray ionization (ESI) source. Separation was carried out on a C18 BEH column (100 × 2.1 mm, 1.7 μm particle size) at 40 °C. Mobile phase was acetonitrile with 0.1% FA (B) and water with 0.1% FA (A) at a flow rate of 0.2 mL/min. Gradient elution was as follows: 20% B initial conditions rising to 70% B at 9.50 min, increasing further to 100% B at 10 min, holding until 11 min, dropping back to 20% B at 12 min and holding until 15 min. Injection volume was 5 μL. Samples were kept at 5 °C.

The Q-TOF was operated in positive ESI mode using MS^E^ acquisition, that allows both precursor and product ions to be acquired simultaneously. Parameters were: 3.2 kV capillary voltage, 40 V cone voltage, 80 °C source temperature, 350 °C desolvation temperature, 50 L/h cone gas flow, 600 L/h desolvation gas flow. Argon was used as the collision gas. MS^E^ consisted in 3 functions: the first function used low collision energy 6 V, the second function used a collision energy ramp of 20–50 eV and the third function acquired the lock mass data for online mass calibration. Sodium formate was used as calibration solution over a mass range of *m*/*z* 50–2000. Leucine enkephalin (*m*/*z* 556.2771 for positive electrospray mode) was infused at a flow rate of 20 μL/min at 10 s intervals to correct for mass drifts. Acquisition and processing of MS data was done using MassLynx v 4.1 software (Waters).

### 2.6 Protein phosphatase inhibition assay

Phosphatase enzymes are able to hydrolyze pNPP into p-nitrophenol that can be measured by absorbance detection. The colorimetric assay was performed as described in Sotoud et al. (2013) with some modifications. Briefly, a stock solution of 5 mM pNPP was prepared freshly in a buffer (pH 8.0) containing 50 mM Tris-HCl, 20 mM MgC_2_ and 0.2 mM MnCl_2_. The PP1c from rabbit (specific activity 5000–15,000 U/mg protein) was prepared in a buffer (pH 7.4) containing 50 mM Tris-HCl, 1 mg/mL bovine serum albumin (BSA), 1 mM MnCl_2_ and 2 mM dithiothreitol. One unit hydrolyses 1 nmol of pNPP per minute at pH 7.4 at 30 °C according to the manufacturer's instructions (Sigma-Aldrich, UK). The assay was conducted in flat-bottom 96-well plates. Ten μL of OA (0.05–50,000 nM) or 10 μL of each sample was placed in each well in triplicate and 10 μL of PP1 solution was added. After 4 min incubation time at room temperature the reaction was initiated by addition of 180 uL of pNPP stock solution. The plate was incubated at 37 °C immediately. PP1 activity was determined by measuring the absorbance at 405 nm at 4 min intervals for 40 min using a microplate reader (Epoch, BioTek, UK) and the Gen5 software for data acquisition (BioTek, UK). IC_50_ determinations were calculated using four-parameter, variable slope, non-linear regression analysis (GraphPad Prism, USA).

### 2.7 Toxicity prediction

The toxicity of OA and its TPs was predicted by using the Toxicity Estimation software Tool (TEST, v. 4.2.1) from the US Environmental Protection Agency (USEPA, 2012). This software predicts toxicity values using mathematical models of Quantitative Structure Activity Relationship (QSAR) (Martin, 2016). In this work, the 96 h fathead minnow 50% lethal concentration (LC_50_), the 48 h *Daphnia magna* LC50, the bioaccumulation factor and the developmental toxicity were chosen as toxicological endpoints. Consensus method was selected as toxicity is estimated by taking an average of the predicted toxicities from five different QSAR methodologies (Hierarchical clustering, Single Model, Group Contribution, the Food and Drug Administration (FDA) and Nearest Neighbor).

## 3 Results and discussion

### 3.1 Screening and identification of DSP compounds in *Prorocentrum lima* culture

Many marine toxins have very complex structures that cannot or are prohibitively expensive to be synthesised. On the other hand, the high cost of commercially available toxin standards (naturally produced) make lab-scale studies requiring high amounts (≈3–5 mg) of toxin unfeasible. Several benthic species of the genus *Prorocentrum* have been confirmed to produce DSPs. Therefore, the culture *P. lima* was assessed as a viable source of DSP toxins, in particular OA, for the photocatalysis experiments.

**Fig. 2:**
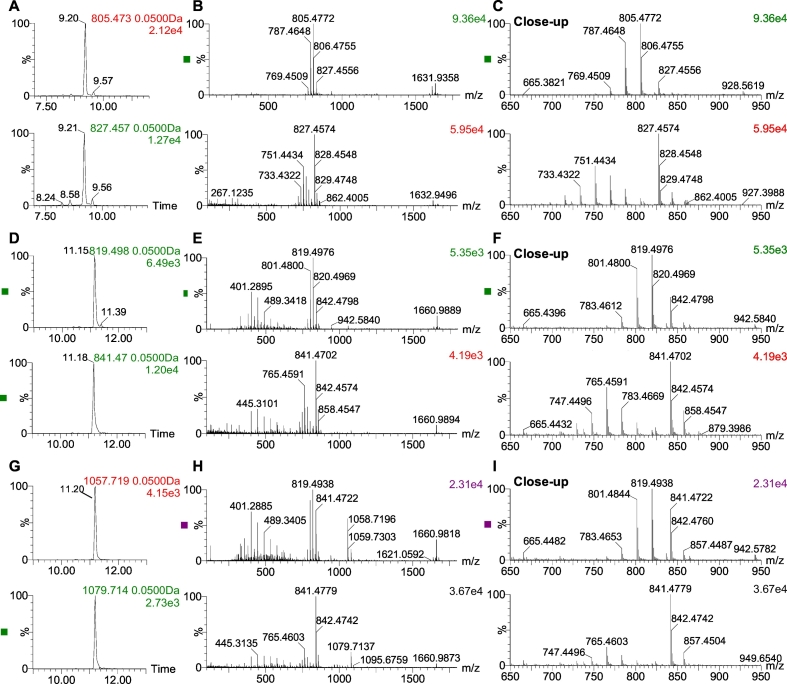
Identification of OA, DTX1 and DTX1 analogue in a *P. lima* culture. Extracted ion chromatogram [M + H]^+^ at 0.05 Da mass window in LE (top) and HE (bottom) for OA (A), DTX1 (D) and DTX1 analogue (G). LE (top) and HE (bottom) TOF mass spectra obtained for OA (B), DTX1 (E) and DTX1 analogue (H). Close up mass spectra of OA (C), DTX1 (F) and DTX1 analogue (I).

As a preliminary screening, extract of *P. lima* culture was analysed using high resolution mass spectrometry in positive mode (Fig. 2). Positive mode MS^E^ fragmentation showed the presence of OA (R_t_ 9.20 min) and DTX1 (R_t_ 11.15 min) in the culture of *P. lima*. Identification was done by comparison of retention time and fragmentation patterns of commercially available standards of OA and DTX1 (Fig. S2) with the samples. The low energy (LE) spectrum (R_t_ 9.20 min) of OA in the *P. lima* culture (Fig. 2) shows the characteristic ions *m*/*z* 805.4772 [M + H]^+^, *m*/*z* 827.4556 [M + Na]^+^, 1631.9358 [2M + Na]^+^, 787.4647 [M + H-H_2_O]^+^. The high energy (HE) spectrum (R_t_ 9.21 min) shows an abundant ion at *m*/*z* 827.4575 [M + Na]^+^, the characteristics losses of water at *m*/*z* 787.4630, 769.4552, 751.4434, 733.4316 and 715.4200 and the specific fragments at *m*/*z* 429.2278, 305.2069 and 287.1950 (Paz et al., 2007; Quilliam, 1995). The LE spectrum of DTX1 (R_t_ 11.15 min) shows the protonated ion *m*/*z* 819.4935, the Na^+^ adduct at *m*/*z* 841.4717, the first loss of H_2_O at *m*/*z* 801.4843 and the multimer Na^+^ adduct at *m*/*z* 1660.9813. The HE (R_t_ 11.16 min) spectrum shows the Na^+^ adduct the consecutives losses of H_2_O at *m*/*z* 801.4865, 783.4685, 765.4592 and 747.4487, and the fragments 267.1255, 301.2088, 401.2854, 445.3135, 489.3397, 533.3653, 577.3853, 621.4148. In addition, a peak coeluted 0.3 min later than DTX1. The LE spectrum (R_t_ 11.20 min) of the coeluting peak showed a signal at *m*/*z* 1057.7203 and 819.4976. No standard was available for comparison but the mass spectra suggested that the coeluting compound is an analogue derived from DTX-1 and maybe a novel diol ester. The LE and HE spectrum showed the following ions *m*/*z*: 819.4938 [DTX1 + H]^+^, 841.4723 [DTX1 + Na]^+^, 801.4844 [DTX1 + H-H_2_O]^+^, 783.4653 [DTX1 + H-2H_2_O]^+^, 765.4743 [DTX1 + H-3H_2_O]^+^, 1659.9741 [2DTX1 + Na]^+^ and the fragments at *m*/*z* 401.2881, 445.3150, 489.3403, 533.3678, 577.3889 (corresponding to losses of -CO_2_) (Carey et al., 2012; Paz et al., 2007; Quilliam, 1995).

Overall, 2 L of concentrated cells contained ≈84 mg of OA, ≈23 mg of DTX1 and ≈17 mg of the analogue derived from DTX1. Toxins were fractionated using a Biotage Selekt Flash Purification System and 28 fractions were obtained. OA was identified by UPLC-QTOF-MS^E^ in fractions 16–19 (60–70% MeOH content), DTX1 in fractions 18–21 (67–77% MeOH content) and DTX1 analogue in fractions 21–23 (77–83% MeOH content). Fraction with the highest purity and content of OA was 17 (>90%, 25.3 mg), for DTX1 fraction 19 (>35%, 11.6 mg) and for DTX1 analogue fraction 22 (>45%, 13.2 mg). *Prorocentrum* species produce different DSP content depending on the species and/or strains (An et al., 2010; Aquino-Cruz et al., 2018; Ben-Gharbia et al., 2016; Lee et al., 2016; Luo et al., 2017; Nishimura et al., 2019; Nishimura et al., 2020; Pan et al., 2017). Previous studies on large-scale culture of *P. lima* and separation and purification of OA and DTX1 were published. For example, Wang et al. (2015) developed a vertical flat plate photobioreactor in which they obtained 15.2 mg/g of OA and 21.6 mg/g of DTX1 from a 60 L culture (3.2 g dry weight) of *P. lima*. Next, Chen et al. (2017) purified these toxins to obtain milligrams of OA and DTX1 of >80% purity using a macroporous resin column and high-speed counter-current chromatography-MS. Further purification with semi-preparative HPLC-MS achieved >98% purity. Although improved purity is obtainable as described by Chen et al. (2017), in our study, the culture of *P. lima* fit the purpose of producing sufficient amounts of OA (≈84 mg) that can be easily isolated and purified (>90%) to be used in photocatalysis studies using a more simple extraction and purification process.

### 3.2 Photocatalytic degradation

#### 3.2.1 Degradation of OA by UV/TiO_2_

OA showed little change in methanolic solutions or deionized water at different temperatures (−20 °C, 4 °C, 20–22 °C) over time (42 days) (Fig. S3). Heating to 45 °C also resulted in no losses of the toxin. OA was found to be very stable. A similar outcome was reported in previous studies. Blanco et al. (2018) reported the high stability of OA in seawater, interstitial water and sediments over a 23-day period. They said that even assuming the highest degradation rate, half of the toxin would remain in water after more than one year after a *Dinophysis* bloom.

As a result of the persistence of the free OA toxin in water bodies, lab-scale photocatalysis (UV-TiO_2_) experiments were performed to explore the effectiveness of this technique to degrade the toxin and subsequently its potential application in the marine environment.

**Fig. 3:**
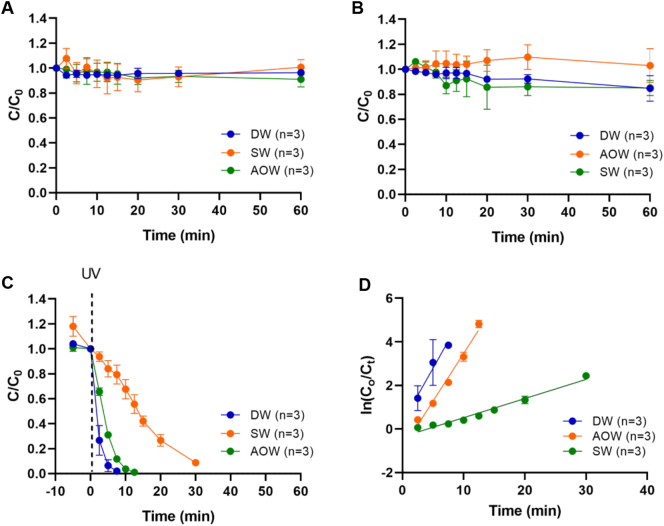
Degradation of OA in deionized water (DW), artificial ocean water (AOW) and seawater (SW) under dark conditions (A), UV irradiation (B) and UV/TiO_2_ (0.1% *w*/*v*) system (C). Pseudo-first order kinetic model fit (D). C_0_: concentration at initial time; C: concentration at specific time. Data is presented as mean values and SD of *n* = 3.

Changes in concentration of OA with different exposure times were plotted in Fig. 3A–B. Results for dark (Fig. 3A) and photolysis (UV irradiation only) (Fig. 3B) indicate that there was little to no reductions in concentrations under experimental conditions after 60 min, which indicates that OA was not adsorbed onto the catalyst or the reactor vessel and that OA is resistant to UV degradation (OA removal: ˂5% in AOW and ˂15% in DW and SW). pH greatly affects the ionization of OA and the surface charge of TiO_2_ which result in a higher or lower adsorption capacity. Within the pH range of the samples here used (6.9–7.8) both OA (pKa 3.87) and the TiO_2_ surface (point of zero charge is pH 6.5) carry negative charges and adsorption on the catalyst surface is suppressed. On the other hand, it was found that OA was completely removed after 7.5 min, 12.5 min and 30 min in DW, AOW and SW, respectively using UV/TiO_2_ 0.1% *w*/*v* (Fig. 3C).

The kinetics of the OA degradation in AOW and SW fitted well to the pseudo-first-order model (R^2^ > 0.96) (Fig. 3D): ln(C_o_/C_t_) = *k* t; where C_o_ and C_t_ are the concentrations of OA in the solution at time 0 and t (min), respectively, *k* is the rate constant (min^−1^). The rate constant was 0.437 min^−1^ in AOW and five times lower in SW (0.088 min^−1^). In the case of DW, due to the fast degradation of OA, there was only three time points to calculate the rate constant so the uncertainty of the results is high. The rate constant in DW was 0.498 min^−1^ at with this pseudo-first-order model but the R^2^ was 0.70 (Fig. 3D). Similar results were obtained when the initial concentration of OA was 10 times lower (1 μg/mL) in DW (0.568 min^−1^, R^2^ > 0.72). Interestingly DW and AOW showed similar rate constants, despite the amount of salts present in AOW. In photocatalytic processes the presence of ions has been reported as a negative effect, at concentrations as low as 10^−3^ M, due to competitive adsorption at the active sites of the catalyst (Mills and Le Hunte, 1997) or acting as radical scavengers (Bennedsen et al., 2012). However, this effect was not observed in this study. There are no previous reports about the photocatalysis effectiveness of UV/TiO_2_ on OA in seawater for comparison. The slower rate constant in SW compared to AOW might be due to the presence of organic matter in the matrix. As it has been reported previously, the nature and concentration of organic matter in the matrix can inhibit or promote photocatalytic reactions (Li and Hu, 2016). In the study of Muff et al. (2017) the influence of the seawater matrix on the photolytic and photocatalytic degradation of an organotin pesticide (tributyltin) was evaluated. They observed a reduction of 41% in seawater compared to demineralized water. Moreover, the photocatalytic TiO_2_ surface was inactivated and produced radicals were scavenged by the relatively high salinity and content of organic matter in the water.

Barriers to real-life application of this technology remain but recent improvements are overcoming them (e.g. UV-LED and solar irradiation as energy sources, new catalyst supporters, modifications of the catalyst to increase the photocatalytic activity). Pestana et al., 2014, Pestana et al., 2020 demonstrated the feasibility of the technology in the field. A packed-bed flow-through UV photocatalytic reactor with pelletised TiO_2_ successfully removed freshwater cyanotoxins and improved overall quality of waste lagoon water (Pestana et al., 2020) as well as it removed trace water contaminants (geosmin and 2-methylisoborneol) from water in a fish-farm raceway (Pestana et al., 2014).

#### 3.2.2 Formation of TPs by UV/TiO_2_

**Fig. 4:**
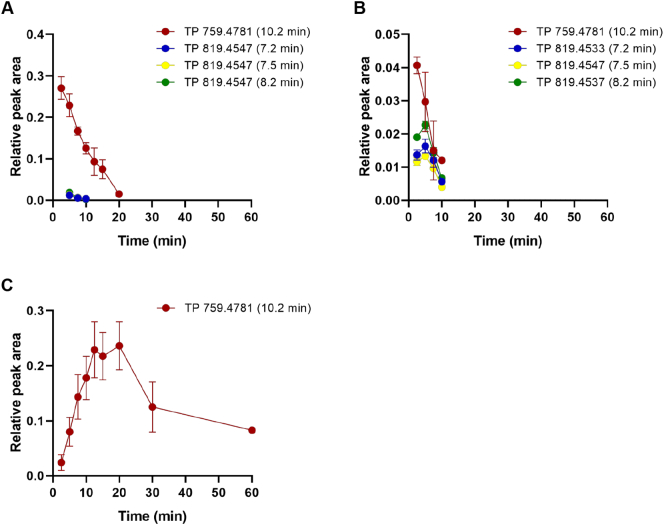
Degradation of transformation products (TPs) formed during UV/TiO_2_ in deionized water (A), artificial ocean water (B) and seawater (C). Data is presented as mean values and SD of n = 3.

Transformation products (TPs) were formed and removed during the photocatalytic degradation of OA using UV/TiO_2_. Overall, four peaks were observed at t_R_ 7.2, 7.5, 8.1 and 10.2 min. Time-dependent evolution profiles of TPs during UV/TiO_2_ processes are shown in Fig. 4. The peak area of each TP was normalized to the initial peak area of OA for semi-quantification due to the lack of analytical standards for their quantitation. TPs arised at the beginning of irradiation and they were found at 2.5 min in all three matrices but were removed after 10 min of irradiation in DW, 20 min in AOW and they were present after 60 min in SW. The peak at 10.2 min was found the most abundant during the photocatalytic process, especially in AOW and SW. This peak was found at its maximum level at 2.5 min in DW and AOW and was removed after 20 and 10 min, respectively. It took longer to reach its maximum level in SW (10–20 min) and there was still 35% present after 60 min of UV irradiation.

Identification of TPs was based on the use of accurate mass measurements and MS^E^ fragmentation (Fig. S4). The peak at 10.2 min with a protonated *m*/*z* of 759.4781 differs in 45 Da with OA (*m*/*z* 805.4775). The tentative identification points to norokadanone generated by the decarboxylation of OA. The spectrum (Fig. S4) shows the characteristic losses of water (*m*/*z* 741.4662, 723.4518, 705.4407), the Na^+^ adduct (*m*/*z* 781.4587) and the multimer 2M + NH_4_^+^ (*m*/*z* 1534.9861). The peaks eluting at 7.2, 7.5 and 8.2 min are minor compounds and share the same spectra (*m*/*z* 819.4547 [M + H]^+^). Although OA methyl ester and 35S DTX1 are isomers the LE and HE spectra show characteristic ions of DTX1 (*m*/*z* 841.4322, 836.4773, 819.4553, 801.4553, 783.4299) that indicates that they could be isomers of DTX1 (Fig. S4) (Pan et al., 2017; Paz et al., 2007).

#### 3.2.3 Toxicity evaluation – phosphatase inhibition assay

Finally, it is important to determine if the photocatalytic system was able to detoxify the seawater. OA and DTxs are potent serine/threonine protein phosphatase inhibitor, for that purpose, the phosphatase inhibition assay was carried out.

The dose-dependent kinetic activity of PP1 in the presence of pNPP (5 mM) is shown in Fig. S5. The optical density (OD) increased linearly with increasing phosphatase concentrations (Fig. S5A) and with time at a fixed concentration of PP1 (5 μg/mL) (Fig. S5B). The concentration-inhibition curve of OA (0–50,000 nM) in the presence of PP1 (5 μg/mL) is shown in Fig. S5C. Under these conditions, the IC_50_ value of OA was 1183 nM, which is higher than the ones reported earlier (IC_50_ 3.6–315 nM) (Bialojan and Takai, 1988; Huhn et al., 2009; Twiner et al., 2016). IC_50_ values are difficult to compare because they depend, among others, on the amount of enzyme and the substrate concentration.

**Fig. 5:**
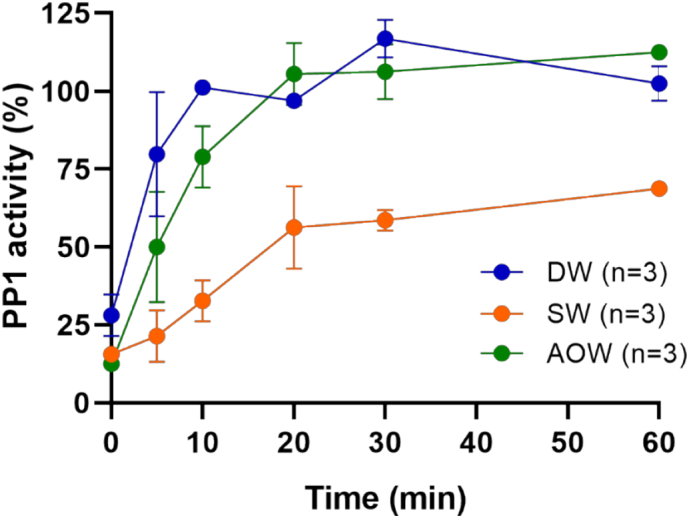
Toxicity profile of OA based on phosphate inhibition measurements in deionized water (DW), artificial ocean water (AOW) and seawater (SW). Data is presented as mean values and SD of n = 3.

The potential toxicity of the samples before and during the heterogeneous photocatalytic degradation of OA was evaluated by the phosphatase inhibition assay. Results are presented as percentage of activity of PP1 in Fig. 5. A 10 μg/mL OA solution exhibited an activity value of 28% in DW, 13% in AOW and 34% in SW. The activity rose as the concentration of OA decreased. It is noteworthy that the highest toxicity was observed at the beginning of the experiment where OA was present at its maximum concentration. According to the above, the higher toxicity at t_0_ could not be ascribed to the formation of toxic intermediates which appeared afterwards. At 10 and 20 min, complete detoxification of the irradiated solution was achieved in DW and AOW, respectively, demonstrating the efficiency of heterogeneous photocatalytic oxidation in the elimination of acute toxicity of OA under the investigated conditions. On the other hand, at 60 min, PP1 activity was 64% in SW when the investigated compounds were removed after 30 min.

Findings from Twiner et al. (2016) demonstrated that small variations at the head region (i.e., C1/C2) of the OA/DTX structures results in significant changes in the phosphatase inhibitory potency. On the other hand, changes in methylation at the tail region (C31 and C35) only have mild effects in toxicological potency. In the present study, toxicity was also predicted by using the Toxicity Estimation Software Tool (TEST, version 4.2.1) from the US Environmental Protection Agency (USEPA, 2012). Compared to OA, the toxicity of DTX1 and TPs was lower.

It is important to note that not all the DSP toxins, even when they are structurally related, show the same biological negative effects and the mode of action is still not clear. For example, it was reported that phosphatase inhibition was responsible for the diarrhetic events (Cohen et al., 1990). However, other reports showed that other actions, such as changes in cytoskeletal elements (Creppy et al., 2002) and or neurotransmitters (Louzao et al., 2015) might be associated to gastrointestinal effects. Methyl okadaate has a higher potency than OA to disrupt the cytoskeleton and it is a non-phosphatase inhibitor (Espiña et al., 2010). Therefore, mechanism of toxicity of DSP toxins must be re-evaluated (Abal et al., 2018; Botana et al., 2016; Valdiglesias et al., 2013).

## 4 Conclusions

*Prorocentrum lima* proved to be a viable source of DSP toxins, in particular OA. Two liters of concentrated cells contained 84 mg of OA, 23 mg of DTX1 and 17 mg of the analogue derived from DTX1. OA could be easily isolated and purified. This is the first study that assessed the effectiveness of UV/TiO_2_ system to degrade OA. The marine toxin was completely degraded after 30 min in seawater and even faster in deionized water (7.5 min). The degradation follows a pseudo-first order kinetic type. Four TPs, norokadanone and three possible isomers of DTX1, were identified in the photocatalytic UV/TiO_2_ system. Detoxification was parallel to OA degradation in deionized and artificial ocean water but not for seawater.

Overall, results suggest that UV/TiO_2_ photocatalysis can be an effective approach for degrading OA and their TPs in the marine environment. Further research should be conducted to look for less energy demanding system that could be easily scaled up. It would be worthy to explore: (i) UV-LED as they can provide energy saving, long lifetime, and environment-friendliness (Hg free); (ii) doped TiO_2_; (iii) different supporters for the catalyst; (iv) solar light activated materials; (v) degradation of intracellular and extracellular toxins.

## CRediT authorship contribution statement

**Dolores Camacho-Muñoz:** Conceptualization, Methodology, Validation, Formal analysis, Investigation, Writing - original draft, Writing - review & editing. **Linda Ann Lawton:** Conceptualization, Methodology, Validation, Resources, Writing - review & editing, Funding acquisition. **Christine Edwards:** Conceptualization, Methodology, Validation, Resources, Writing - review & editing, Funding acquisition.

## Declaration of competing interest

The authors declare that they have no known competing financial interests or personal relationships that could have appeared to influence the work reported in this paper.

## Appendix A Supplementary data

Supplementary material Image 1

## References

[bb0005] 225/EC (2002). Commission Decision of 15 March 2002 Laying Down Detailed Rules for the Implementation of Council Directive 91/492/EEC as Regards the Maximum Levels and the Methods of Analysis of Certain Marine Biotoxins in Bivalve Molluscs, Echinoderms, Tunicates and Marine Gastropods (Text With EEA Relevance) (Notified Under Document Number C(2002) 1001).

[bb0010] Abal P., Louzao M.C., Suzuki T., Watanabe R., Vilariño N., Carrera C., Botana A.M., Vieytes M.R., Botana L.M. (2018). Toxic action reevaluation of okadaic acid, dinophysistoxin-1 and dinophysistoxin-2: toxicity equivalency factors based on the oral toxicity study. Cell. Physiol. Biochem..

[bb0015] An T., Winshell J., Scorzetti G., Fell J.W., Rein K.S. (2010). Identification of okadaic acid production in the marine dinoflagellate Prorocentrum rhathymum from Florida Bay. Toxicon.

[bb0020] Anderson C.R., Berdalet E., Kudela R.M., Cusack C.K., Silke J., O'Rourke E., Dugan D., McCammon M., Newton J.A., Moore S.K., Paige K., Ruberg S., Morrison J.R., Kirkpatrick B., Hubbard K., Morell J. (2019). Scaling up from regional case studies to a global harmful algal bloom observing system. Front. Mar. Sci..

[bb0025] Antoniou M.G., Boraei I., Solakidou M., Deligiannakis Y., Abhishek M., Lawton L.A., Edwards C. (2018). Enhancing photocatalytic degradation of the cyanotoxin microcystin-LR with the addition of sulfate-radical generating oxidants. J. Hazard. Mater..

[bb0030] Aquino-Cruz A., Purdie D.A., Morris S. (2018). Effect of increasing sea water temperature on the growth and toxin production of the benthic dinoflagellate Prorocentrum lima. Hydrobiologia.

[bb0035] Ben-Gharbia H., Yahia O.K.-D., Amzil Z., Chomérat N., Abadie E., Masseret E., Sibat M., Zmerli Triki H., Nouri H., Laabir M. (2016). Toxicity and growth assessments of three thermophilic benthic dinoflagellates (*Ostreopsis* cf. *ovata, Prorocentrum lima* and *Coolia monotis*) developing in the southern Mediterranean Basin. Toxins.

[bb0040] Bennedsen L.R., Muff J., Søgaard E.G. (2012). Influence of chloride and carbonates on the reactivity of activated persulfate. Chemosphere.

[bb0045] Bialojan C., Takai A. (1988). Inhibitory effect of a marine-sponge toxin, okadaic acid, on protein phosphatases. Specificity and kinetics. Biochem. J..

[bb0050] Blanco J., Martín-Morales E., Álvarez G. (2018). Stability of okadaic acid and 13-desmethyl spirolide C in seawater and sediment. Mar. Chem..

[bb0055] Bosch-Orea C., Sanchís J., Farré M., Barceló D. (2017). Analysis of lipophilic marine biotoxins by liquid chromatography coupled with high-resolution mass spectrometry in seawater from the Catalan Coast. Anal. Bioanal. Chem..

[bb0060] Botana L.M., Alfonso A., Rodríguez I., Botana A.M., Louzao M.D.C., Vieytes M.R. (2016). How safe is safe for marine toxins monitoring?. Toxins.

[bb0065] Bravo I., Fernández M.L., Ramilo I., Martínez A. (2001). Toxin composition of the toxic dinoflagellate Prorocentrum lima isolated from different locations along the Galician coast (NW Spain). Toxicon.

[bb0070] Campbell K., Vilariño N., Botana L.M., Elliott C.T. (2011). A European perspective on progress in moving away from the mouse bioassay for marine-toxin analysis. TrAC Trends Anal. Chem..

[bb0075] Carey B., Sáez M.J.F., Hamilton B., O'Halloran J., van Pelt F.N.A.M., James K.J. (2012). Elucidation of the mass fragmentation pathways of the polyether marine toxins, dinophysistoxins, and identification of isomer discrimination processes. Rapid Commun. Mass Spectrom..

[bb0080] Chen J., Wang Y., Pan L., Shen H., Fu D., Fu B., Sun C., Zheng L. (2017). Separation and purification of two minor typical diarrhetic shellfish poisoning toxins from harmful marine microalgae via combined liquid chromatography with mass spectrometric detection. J. Sep. Sci..

[bb0085] Cohen P., Holmes C.F.B., Tsukitani Y. (1990). Okadaic acid: a new probe for the study of cellular regulation. Trends Biochem. Sci..

[bb0090] Creppy E.E., Traoré A., Baudrimont I., Cascante M., Carratú M.-R. (2002). Recent advances in the study of epigenetic effects induced by the phycotoxin okadaic acid. Toxicology.

[bb0095] Dell'Aversano C., Tartaglione L. (2017). Mass spectrometry–based methods for the structural characterization of marine toxins. Compr. Anal. Chem..

[bb0100] Djaoued Y., Thibodeau M., Robichaud J., Balaji S., Priya S., Tchoukanova N., Bates S.S. (2008). Photocatalytic degradation of domoic acid using nanocrystalline TiO_2_ thin films. J. Photochem. Photobiol. A Chem..

[bb0105] Dounay A.B., Forsyth C.J. (2002). Okadaic acid: the archetypal serine/threonine protein phosphatase inhibitor. Curr. Med. Chem..

[bb0110] Espiña B., Louzao M., Cagide E., Alfonso A., Vieytes M.R., Yasumoto T., Botana L.M. (2010). The methyl ester of okadaic acid is more potent than okadaic acid in disrupting the actin cytoskeleton and metabolism of primary cultured hepatocytes. Br. J. Pharmacol..

[bb0115] Farabegoli F., Blanco L., Rodríguez L.P., Vieites J.M., Cabado A.G. (2018). Phycotoxins in marine shellfish: origin, occurrence and effects on humans. Marine Drugs.

[bb0120] Fux E., Bire R., Hess P. (2009). Comparative accumulation and composition of lipophilic marine biotoxins in passive samplers and in mussels (*M. edulis*) on the West Coast of Ireland. Harmful Algae.

[bb0125] Gerssen A., Pol-Hofstad I.E., Poelman M., Mulder P.P.J., Van den Top H.J., De Boer J. (2010). Marine toxins: chemistry, toxicity, occurrence and detection, with special reference to the Dutch situation. Toxins.

[bb0130] Guillard R.R.L., Ryther J.H. (1962). Studies of marine planktonic diatoms: I. Cyclotella nana Hustedt, and Detonula confervacea (Cleve) Gran. Can. J. Microbiol..

[bb0135] Hattenrath-Lehmann T.K., Lusty M.W., Wallace R.B., Haynes B., Wang Z., Broadwater M., Deeds J.R., Morton S.L., Hastback W., Porter L., Chytalo K., Gobler C.J. (2018). Evaluation of rapid, early warning approaches to track shellfish toxins associated with *Dinophysis* and *Alexandrium* blooms. Marine Drugs.

[bb0140] He X., Chen J., Wu D., Wang J., Xin M., Liu L., Sun P., Wang B. (2020). Occurrence, distribution, source, and influencing factors of lipophilic marine algal toxins in Laizhou Bay, Bohai Sea, China. Mar. Pollut. Bull..

[bb0145] Huhn J., Jeffrey P.D., Larsen K., Rundberget T., Rise F., Cox N.R., Arcus V., Shi Y., Miles C.O. (2009). A structural basis for the reduced toxicity of dinophysistoxin-2. Chem. Res. Toxicol..

[bb0150] Ibhadon A.O., Fitzpatrick P. (2013). Heterogeneous photocatalysis: recent advances and applications. Catalysts.

[bb0155] Kantiani L., Llorca M., Sanchís J., Farré M., Barceló D. (2010). Emerging food contaminants: a review. Anal. Bioanal. Chem..

[bb0160] Khan U., Benabderrazik N., Bourdelais A.J., Baden D.G., Rein K., Gardinali P.R., Arroyo L., O'Shea K.E. (2010). UV and solar TiO_2_ photocatalysis of brevetoxins (PbTxs). Toxicon.

[bb0165] Kinley C.M., Hendrikse M., Calomeni A.J., Geer T.D., Rodgers J.H. (2018). Solar photocatalysis using fixed-film TiO_2_ for microcystins from colonial Microcystis aeruginosa. Water Air Soil Pollut..

[bb0170] Lee T.C.-H., Fong F.L.-Y., Ho K.-C., Lee F.W.-F. (2016). The mechanism of diarrhetic shellfish poisoning toxin production in Prorocentrum spp.: physiological and molecular perspectives. Toxins.

[bb0175] Li S., Hu J. (2016). Photolytic and photocatalytic degradation of tetracycline: effect of humic acid on degradation kinetics and mechanisms. J. Hazard. Mater..

[bb0180] Liu I., Lawton L.A., Bahnemann D.W., Liu L., Proft B., Robertson P.K.J. (2009). The photocatalytic decomposition of microcystin-LR using selected titanium dioxide materials. Chemosphere.

[bb0185] Louzao M.C., Fernández D.A., Abal P., Fraga M., Vilariño N., Vieytes M.R., Botana L.M. (2015). Diarrhetic effect of okadaic acid could be related with its neuronal action: changes in neuropeptide Y. Toxicol. Lett..

[bb0190] Luo Z., Zhang H., Krock B., Lu S., Yang W., Gu H. (2017). Morphology, molecular phylogeny and okadaic acid production of epibenthic Prorocentrum (Dinophyceae) species from the northern South China Sea. Algal Res..

[bb0195] Martin T.M. (2016). User's Guide for T.E.S.T. (Version 4.2) (Toxicity Estimation Software Tool).

[bb0200] Matafonova G., Batoev V. (2018). Recent advances in application of UV light-emitting diodes for degrading organic pollutants in water through advanced oxidation processes: a review. Water Res..

[bb0205] Mills A., Le Hunte S. (1997). An overview of semiconductor photocatalysis. J. Photochem. Photobiol. A Chem..

[bb0210] Muff J., Simonsen M.E., Søgaard E.G. (2017). Removal of tributyltin from contaminated seawater by combinations of photolytic and TiO_2_ mediated photocatalytic processes. Journal of Environmental Chemical Engineering.

[bb0215] Munday R. (2013). Is protein phosphatase inhibition responsible for the toxic effects of okadaic acid in animals?. Toxins.

[bb0220] Nishimura T., Uchida H., Noguchi R., Oikawa H., Suzuki T., Funaki H., Ihara C., Hagino K., Arimitsu S., Tanii Y., Abe S., Hashimoto K., Mimura K., Tanaka K., Yanagida I., Adachi M. (2019). Abundance of the benthic dinoflagellate Prorocentrum and the diversity, distribution, and diarrhetic shellfish toxin production of Prorocentrum lima complex and P. caipirignum in Japan. Harmful Algae.

[bb0225] Nishimura T., Uchida H., Suzuki T., Tawong W., Abe S., Arimitsu S., Adachi M. (2020). First report on okadaic acid production of a benthic dinoflagellate Prorocentrum cf. fukuyoi from Japan. Phycol. Res..

[bb0230] Pan L., Chen J., Shen H., He X., Li G., Song X., Zhou D., Sun C. (2017). Profiling of extracellular toxins associated with diarrhetic shellfish poison in Prorocentrum lima culture medium by high-performance liquid chromatography coupled with mass spectrometry. Toxins.

[bb0235] Paz B., Daranas A.H., Cruz P.G., Franco J.M., Pizarro G., Souto M.L., Norte M., Fernández J.J. (2007). Characterisation of okadaic acid related toxins by liquid chromatography coupled with mass spectrometry. Toxicon.

[bb0240] Pestana C.J., Robertson P.K.J., Edwards C., Wilhelm W., McKenzie C., Lawton L.A. (2014). A continuous flow packed bed photocatalytic reactor for the destruction of 2-methylisoborneol and geosmin utilising pelletised TiO_2_. Chem. Eng. J..

[bb0245] Pestana C.J., Edwards C., Prabhu R., Robertson P.K.J., Lawton L.A. (2015). Photocatalytic degradation of eleven microcystin variants and nodularin by TiO_2_ coated glass microspheres. J. Hazard. Mater..

[bb0250] Pestana C.J., Hobson P., Robertson P.K.J., Lawton L.A., Newcombe G. (2020). Removal of microcystins from a waste stabilisation lagoon: evaluation of a packed-bed continuous flow TiO_2_ reactor. Chemosphere.

[bb0255] Pinho L.X., Azevedo J., Brito Â., Santos A., Tamagnini P., Vilar V.J.P., Vasconcelos V.M., Boaventura R.A.R. (2015). Effect of TiO_2_ photocatalysis on the destruction of Microcystis aeruginosa cells and degradation of cyanotoxins microcystin-LR and cylindrospermopsin. Chem. Eng. J..

[bb0260] Pizarro G., Paz B., González-Gil S., Franco J.M., Reguera B. (2009). Seasonal variability of lipophilic toxins during a Dinophysis acuta bloom in Western Iberia: differences between picked cells and plankton concentrates. Harmful Algae.

[bb0265] Praptiwi R.A. (2014). Optimisation of High Value Metabolite Production From Benthic Marine Dinoflagellate *Prorocentrum lima*. https://rgu-repository.worktribe.com/output/248479/optimisation-of-high-value-metabolite-production-from-benthic-marine-dinoflagellate-prorocentrum-lima.

[bb0270] Quilliam M.A. (1995). Analysis of diarrhetic shellfish poisoning toxins in shellfish tissue by liquid chromatography with fluorometric and mass spectrometric detection. J. AOAC Int..

[bb0275] Reguera B., Riobó P., Rodríguez F., Díaz P.A., Pizarro G., Paz B., Franco J.M., Blanco J. (2014). *Dinophysis* toxins: causative organisms, distribution and fate in shellfish. Marine Drugs.

[bb0280] Rossignoli A.E., Fernández D., Regueiro J., Mariño C., Blanco J. (2011). Esterification of okadaic acid in the mussel Mytilus galloprovincialis. Toxicon.

[bb0285] Schneider O.M., Liang R., Bragg L., Jaciw-Zurakowsky I., Fattahi A., Rathod S., Peng P., Servos M.R., Zhou Y.N. (2019). Photocatalytic degradation of microcystins by TiO_2_ using UV-LED controlled periodic illumination. Catalysts.

[bb0290] Shen L., Xu H., Guo X. (2012). Satellite remote sensing of harmful algal blooms (HABs) and a potential synthesized framework. Sensors.

[bb0295] Shephard G.S., Stockenström S., de Villiers D., Engelbrecht W.J., Wessels G.F.S. (2002). Degradation of microcystin toxins in a falling film photocatalytic reactor with immobilized titanium dioxide catalyst. Water Res..

[bb0300] Sotoud H., Gribbon P., Ellinger B., Reinshagen J., Boknik P., Kattner L., El-Armouche A., Eschenhagen T. (2013). Development of a colorimetric and a fluorescence phosphatase-inhibitor assay suitable for drug discovery approaches. J. Biomol. Screen..

[bb0305] Suzuki T., Ota H., Yamasaki M. (1999). Direct evidence of transformation of dinophysistoxin-1-7-*O*-acyl-dinophysistoxin-1 (dinophysistoxin-3) in the scallop *Patinopecten yessoensis*. Toxicon.

[bb0310] Twiner M.J., Doucette G.J., Pang Y., Fang C., Forsyth C.J., Miles C.O. (2016). Structure–activity relationship studies using natural and synthetic okadaic acid/dinophysistoxin toxins. Marine Drugs.

[bb0315] Uchida H., Watanabe R., Matsushima R., Oikawa H., Nagai S., Kamiyama T., Baba K., Miyazono A., Kosaka Y., Kaga S., Matsuyama Y., Suzuki T. (2018). Toxin profiles of okadaic analogues and other lipophilic toxins in *Dinophysis* from Japanese coastal waters. Toxins.

[bb4000] USEPA (2012). Toxicity Estimation Software Tool (TEST). https://www.epa.gov/chemical-research/toxicity-estimation-software-tool-tes.

[bb0320] Valdiglesias V., Prego-Faraldo M.V., Pásaro E., Méndez J., Laffon B. (2013). Okadaic acid: more than a diarrheic toxin. Marine Drugs.

[bb0325] Vale P. (2010). Profiles of fatty acids and 7-O-acyl okadaic acid esters in bivalves: can bacteria be involved in acyl esterification of okadaic acid?. Comparative Biochemistry and Physiology Part C: Toxicology & Pharmacology.

[bb0330] Wang S., Chen J., Lii Z., Wang Y., Fu B., Han X., Li Z. (2015). Cultivation of the benthic microalga *Prorocentrum lima* for the production of diarrhetic shellfish poisoning toxins in a vertical flat photobioreactor. Bioresour. Technol..

