# Supplementary material for: Degradation of okadaic acid in seawater by UV/TiO2 photocatalysis – Proof of concept
Source: Sci Total Environ. 2020 Sep 1;733:139346. doi: 10.1016/j.scitotenv.2020.139346 (PMC7298613; doi:10.1016/j.scitotenv.2020.139346)
Supplement: Supplementary file 1 — Supplementary material [file mmc1.docx]

**Supplementary material**

Fig. S1. Structures of okadaic acid and dinophysistoxin 1 (DTX1)

Figure S2. MS data of OA and DTX1 standards. Extracted ion chromatogram [M+H]^+^ at 0.05 Da mass window in LE (top) and HE (bottom) for OA (A) and DTX1 (D). LE (top) and HE (bottom) TOF mass spectra obtained for OA (B) and DTX1 (E). Close up mass spectra of OA (C) and DTX1 (F).


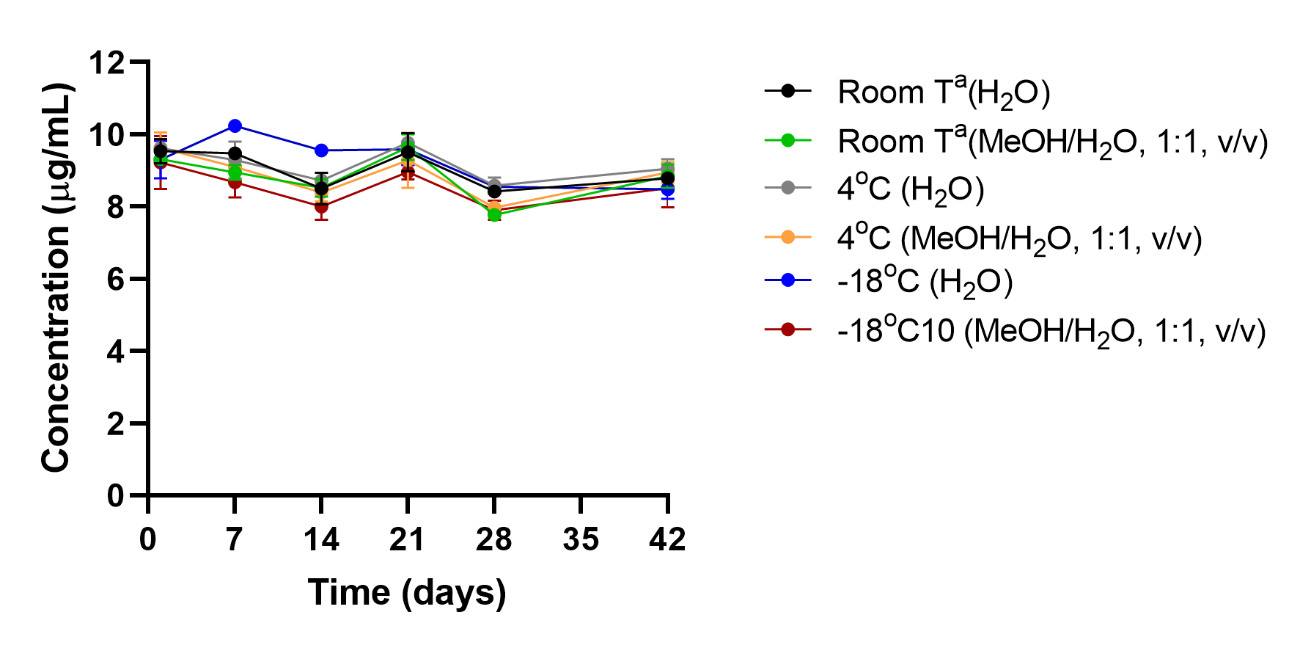


Figure S3. Effect of temperature and nature of solvent in stability of OA.

Figure S4. MS data of transformation products generated during UV/TiO_2_ system. Extracted ion chromatogram [M+H]^+^ at 0.05 Da mass window in LE (top) and HE (bottom) for suspected norokadanone (A) and isomers of DTX1 (D). LE (top) and HE (bottom) TOF mass spectra obtained for norokadanone (B) and isomers of DTX1 (E). Close up mass spectra of norokadanone (C) and isomers of DTX1 (F).

Figure S5. Dose dependent kinetic activity of PP1 in the presence of pNPP (5 mM) (A, B). Optical density (OD) was measured at 405 nm. Effect of OA concentration on PP1 (5 µg/mL) (C). Three-parameter, variable slope, non-linear dose response analysis was performed and calculated concentration of inhibition at 50% (IC_50_). Data is expressed as mean values and SD of n=3.

Table S1. Toxicological values of okadaic acid (OA), dinophysistoxin 1 (DTX1) and norokadanone during photocatalysis predicted by the USEPA TEST

|  | **Biaccumulation factor** | **Developmental toxicity value** | **Daphnia magna LC50 (48 h) (mg/L)** | **Fathead minnow LC50 (96 h) (mg/L)** |
| --- | --- | --- | --- | --- |
| OA | 9.17 | 0.70 | 42.1 | 0.003 |
| DTX1 | 4.22 | 0.74 | 29.0 | 0.008 |
| Norokadanone | 1.77 | 0.52 | 32.6 | - |
